# Color Perspectives in Aquatic Explorations: Unveiling Innate Color Preferences and Psychoactive Responses in Freshwater Crayfish

**DOI:** 10.3390/toxics11100838

**Published:** 2023-10-03

**Authors:** Michael Edbert Suryanto, Gilbert Audira, Marri Jmelou M. Roldan, Hong-Thih Lai, Chung-Der Hsiao

**Affiliations:** 1 Department of Chemistry, Chung Yuan Christian University, Taoyuan 320314, Taiwan; michael.edbert93@gmail.com; 2 Department of Bioscience Technology, Chung Yuan Christian University, Taoyuan 320314, Taiwan; gilbertaudira@yahoo.com; 3 Faculty of Pharmacy, The Graduate School, University of Santo Tomas, Manila 1008, Philippines; mmroldan@ust.edu.ph; 4 Department of Aquatic Biosciences, National Chiayi University, 300 University Rd., Chiayi 60004, Taiwan; hongthih@gmail.com; 5 Center for Nanotechnology, Chung Yuan Christian University, Taoyuan 320314, Taiwan; 6 Research Center for Aquatic Toxicology and Pharmacology, Chung Yuan Christian University, Taoyuan 320314, Taiwan

**Keywords:** color preference, crayfish, *P. clarkii*, *C. quadricarinatus*, antidepressant

## Abstract

Color preference assay is a test for an animal’s innate and adaptive response to differentiate colors and can be used as an endpoint for psychoactive activity evaluation. Several color preference test methods in aquatic animals that can be used to perform behavioral screening have been established. However, the color preference test conditions have yet to be extensively studied and standardized in aquatic invertebrates. This study aimed to replicate and optimize the previously published method to evaluate the potential color preference in freshwater crayfish based on four different approaches: species, life stages, sex, and pharmaceutical exposure. Using the optimized setup, two crayfish species display color preferences to some specific colors. *P. clarkii* displays more dominant color preference behavior than *C. quadricarinatus* in terms of color preference ranking and index. *P. clarkii* prefers the red color compared to other colors (red > green > blue > yellow), while *C. quadricarinatus* dislikes yellow compared to other colors (blue = green = red > yellow). Since *P. clarkii* has a more obvious color index ranking and several advantages compared to *C. quadricarinatus*, we conducted further tests using *P. clarkii* as an animal model. In the juvenile and adult stages of *P. clarkii*, they prefer red and avoid yellow. However, the juvenile one did not display a strong color preference like the adult one. Different sex of crayfish displayed no significant differences in their color preference responses. In addition, we also evaluated the potential effect of the antidepressant sertraline on color preference in *P. clarkii* and found that waterborne antidepressant exposure can significantly alter their color preference. This fundamental information collected from this study supports the crayfish color preference test as a good behavioral test to address environmental pollution.

## 1. Introduction

Color vision is essential for animals to survive. It plays a critical role in visual perception and aids the animals’ responses toward environmental stimuli [1]. It helps them locate food, shelter, mates, recognize predators, and navigate. This color perception is an innate [2] and adaptive response [3]. Animals may display an innate color preference for certain types of colors that match their environment or the resources in the environment [4]. A previous study showed that guppies (*Poecilia reticulata*) are attracted to the orange color in the mating or feeding context [5]. Different environmental colors also affect the vision of fish, influencing their food intake and stress response, then further affecting their growth and fitness [6]. Under fish farm conditions, Nile tilapia were reared to a new environment and adapted to a blue color preference, resulting in an optimal rearing environment [7]. It was reported that the fish had an adaptive response toward color, and the adaptation took around 14 days. The color preference ranking displays ontogenic change over time. For example, zebrafish (*Danio rerio*) larvae at five days post fertilization (5 dpf) preferred blue over other colors (blue > red > green > yellow), with blue being the most favorable color and yellow being the most avoided color. However, the color preference ranking is shifted to red > blue > green > yellow at the adult stage, which was reported by Siregar et al., 2020 [8]. The color preference also displays species-specific differences in fish. For example, the innate color preference ranking for freshwater fish like tiger barb (*Puntigrus tetrazona*) is green > blue > red > yellow, and for glass catfish (*Kryptopterus vitreolus*) it is red > blue > green > yellow. Like zebrafish, yellow is the least preferred color for tiger barb and glass catfish [8]. Other publications related to innate color preference have been investigated in many animals, including insects (fruit flies, bees, and hawkmoths) [9,10,11], poultry (chickens and ducks) [12], and several different species of frogs and tadpoles [13,14,15,16]. Further evidence of color preference in different animal classes, such as mollusks, spiders, reptiles, birds, and mammals, was described by Kelber et al., 2003 [17]. Innate color preference provides a better understanding of animals’ behaviors and their senses to determine or respond to specific colors adaptively. To see colors, animals rely on the photoreceptor known as cones to distribute the wavelengths of light. Crayfish consists of two compound eyes with photoreceptor neurons sensitive to light [18]. Animals, including humans, can perceive different colors depending on the number of cone types. Humans and some primates have three types of cones (blue, green, and red), called trichromatic vision, that can differentiate visible wavelength. However, lower vertebrates, such as fish, are tetrachromatic, which can distinguish ultraviolet (UV) and visible wavelengths [1]. Meanwhile, for crustaceans, few publications discussed their color preference [19,20,21]. To the best of our knowledge, no publication discusses innate color preference for crayfish (innate color preference in other crustacean species was summarized in Appendix A, Table A1). Since crustaceans have also been identified as having multiple cone types of color receptors, we hypothesize crayfish might also have an innate color preference.

**Figure A1:**
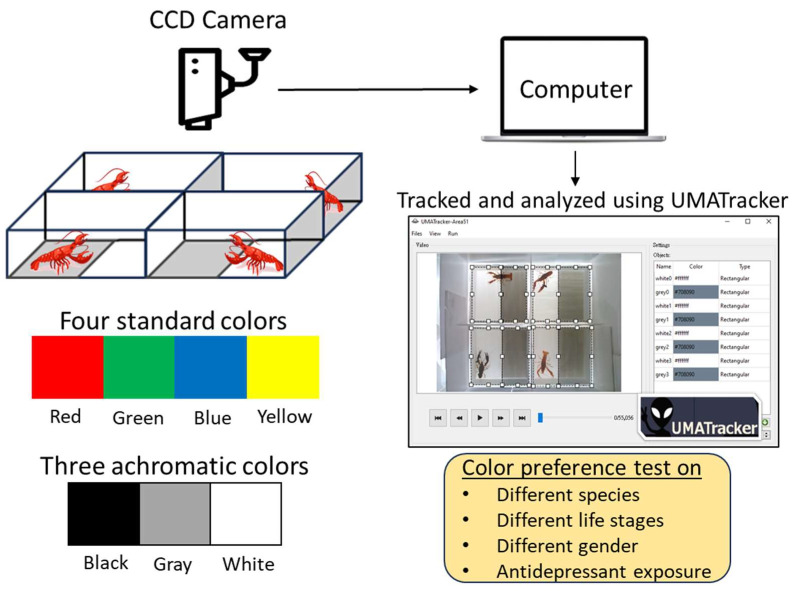
Graphic illustration of entire experimental workflow to study color preference in crayfish.

In this study, we investigate whether crayfish have any color preference or none. We evaluated two different species, the Australian tropical blue crayfish (*Cherax quadricarinatus*) and American red swamp crayfish (*Procambarus clarkii*). *P. clarkii* is the most widespread crayfish species globally, which is very popular for human consumption and aquaria in many countries [22]. *C. quadricarinatus* is also a popular crayfish with several commercial attributes for aquaculture purposes [23]. This species displays high growth rates and ecological tolerance despite the wide variations in water quality. Previous studies displayed the importance of aquarium color background on the crayfish aquaculture. They reported that juvenile crayfish cultured in a dark background exhibited enhanced growth and weight gain [24]. Meanwhile, blue light suppressed the larval growth of red swamp crayfish [25]. Regarding their sexual difference, the growth of females and males were both suppressed by blue light, although the female was more sensitive than the male. Despite their economic benefits, freshwater crayfish are known to become a very invasive species. They are fast-growing and adaptable, bringing adverse impacts and threats on native species and could irreversibly damage their populations. There are numerous reports of crayfish predation on amphibians and fish, with a high consumption rate of their eggs. Crayfish also occupied their habitat, competing for food and shelter, resulting in the decline in amphibian and fish populations [26]. They are also physically destructive, burrowing holes, including damage to environmental substrates that cause harmful effects to the ecosystem. Therefore, environmental control measures are needed to suppress the population of this invasive crayfish. Ahmadi and colleagues developed an enhanced conventional gear, baited traps equipped with an LED light to improve the crayfish harvesting and collecting procedures. This revealed that the crayfish response was significantly increased toward green, blue, and yellow LED light traps [27]. These findings support the importance of our study to investigate the innate color preference of crayfish. Eventually, it will give a better understanding of crayfish dynamic behavior and their responses. We conducted this experiment based on our previously published method, validated as a standardized protocol in zebrafish and other teleost [8]. Four standard colors: red, green, blue, and yellow, as well as achromatic colors: black, gray, and white, were tested. We explored the potential use of the optimized method to examine color preference ranking and index choice differences in four different approaches: species, life stages, sexes, and chemical exposure (workflow and design are summarized in Figure A1, Appendix A).

## 2. Materials and Methods

### 2.1. Animal Husbandry

Both freshwater crayfish, *P. clarkii* and *C. quadricarinatus*, were purchased from a local aquarium store (Zhongli, Taoyuan, Taiwan). They were maintained in our laboratory and acclimatized for 1 month. All animals were housed in a plastic tank individually with systematic water circulation under the lighting room condition with 14 h light and 10 h dark. Adult (3–4 months old) crayfish with total body lengths of 6–7 cm were selected for the color preference test. At first, for the preliminary test, we compared the color preference from these two different species. Then, *P. clarkii* was chosen for further experimentation because its color preference was more consistent and clearer compared to *C. quadricarinatus*. We continued the test in different life stages with 20 juvenile *P. clarkii* crayfish (~1 month old) of random sex to observe whether there is any difference compared to the adults. Juvenile crayfish were purchased from the same local aquarium store. After that, we also conducted a test for specific-sex behavior in male and female adult *P. clarkii* crayfish (20 in each group). Water was changed every two days, and the crayfish were fed commercial shrimp pellets 30 min before each water change. The water was maintained at a temperature of 24 ± 1 °C, with pH of 7.0 ± 0.5 and dissolved oxygen at 6.5 ± 0.2 mg/L.

### 2.2. Antidepressant Exposure

Sertraline is known as a selective serotonin reuptake inhibitor (SSRI). Sertraline hydrochloride was purchased from Shanghai Macklin Biomedical Co., Ltd., Shanghai, China. Sertraline was dissolved in distilled water as 1000× stock and stored at 4 °C. The antidepressant was diluted from the stock solution to a working concentration (1 µg/L). Sertraline was administered to the crayfish via immersion in the water tank. In this experiment, a mixed-sex group of *P. clarkii* (6 males and 6 females) were selected and systematically exposed to 1 µg/L of sertraline for 14 days. The water was refreshed every day during the exchange of exposure solution to maintain the chemical exposure. After 14 days, the color preference assay was conducted.

### 2.3. Instrument Setting used to Measure Achromatic and Color Preference

The instrument setting for the color preference assay was conducted based on the previous method [8]. The assay was conducted in a 21 × 21 × 10 cm acrylic tank filled with 1.5 L of filtered water. Two different position LED lights were used simultaneously to provide uniform illumination from either the top or bottom positions. Four 30 W LED lights were positioned above, and one 60 × 60 cm 24 W LED plate was below the acrylic tanks. Each half of the tank was covered with a combination of two-color plates. A single batch was performed with four sets of acrylic tanks with one crayfish in each tank (four crayfish for each batch). We conducted five batches of a color preference assay in this experiment. We evaluated with a total *N* number = 20 crayfish for each species, *P. clarkii* and *C. quadricarinatus*. Six different combinations of colors were used in this experiment: (1) blue–red; (2) green–blue; (3) green–red; (4) yellow–blue; (5) yellow–green; and (6) yellow–red. We also conducted the crayfish preference assay with achromatic colors to validate the previous literature that displayed the strong preference of crayfish for black or dark environments. We further evaluated this using (7) gray–black; (8) white–black; and (9) white–gray color combinations to display that the environment is not favored by the crayfish. All the color preference assays were conducted at room temperature conditions (24–25 °C). The wavelength (spectrum), reflectance, and irradiance (emission intensity) of all color plates are listed in Table A2.

### 2.4. Video Taping and Trajectory Analysis by using UMATracker

The color preference behavior of crayfish was recorded using a high-quality CCD camera with a maximum resolution of 1920 × 1080 pixels and 30 fps (frame rate per second). The camera was connected to a desktop computer using Debut Video Capture Free Edition (NCH Software, Inc., Greenwood Village, CO, USA, https://www.nchsoftware.com/capture/index.html) (accessed on 5 January 2023). Each batch was recorded with a total duration of 30 min. The crayfish locomotion was then analyzed using an open-source UMATracker software release-15 (Yamanaka and Takeuchi, Hiroshima, Japan, https://ymnk13.github.io/UMATracker/) (accessed on 11 March 2023) [28]. Generally, the video was loaded to the tracker software, and by using the filter generator, we preprocessed the video by removing the background to retrieve and distinguish the animal images. The animal images were then converted to grayscale and binarized by adjusting the threshold value. We obtained the output result of the converted video frame by thresholding the image binarization algorithm. At the tracking step, we set the number of animals in the video frame. Then, run the tracking algorithm to detect the position of each animal. With further analysis using the region of interest (ROI) feature, we could distinguish the area where the animal spent from each frame. Since two rectangular plates with different color plates were used for each tank, we created two ROIs, one each for each color. After successfully analyzing the ROI, the data were saved to .csv file format. These data contain the XY coordinates and animals’ positions per frame, which could be opened using Microsoft Excel 2016 MSO Version 2308 Build 16.0.16731.20182 32-bit (Microsoft Corporation, Redmond, WA, USA).

### 2.5. Calculation of Color Preference Index

The choice index equation was used to calculate the color preferences of crayfish. This index was measured by subtracting time spent in the first color from time spent in second color partition, then divided by the total video time [8,29]. The duration of time spent (s) in each color partition combinations were calculated from three different trials: trial 1 (0–10 min), trial 2 (10–20 min), and trial 3 (20–30 min). The total of time spent was calculated from all these three consecutive trials.

$$Choice\;index=\frac{Time\;spent\;in\;first\;color\;partition\left(s\right)-Time\;spent\;in\;second\;color\;partition(s)}{Total\;video\;time(s)}$$

### 2.6. Statistical Analysis

Graphic results and statistical analysis were conducted using GraphPad Prism 8.02 (GraphPad Software, Inc.: San Diego, CA, USA) (https://www.graphpad.com/) (accessed on 9 May 2023). An unpaired *t*-test was used to analyze the color preference index and compare the significant differences between the two-color combination. The data shown were presented as means ± SEM with *p* < 0.05 regarded as the statistically significant difference at 95% confidence. The significant difference is marked as * if *p* < 0.05, ** if *p* < 0.01, *** if *p* < 0.001, and **** if *p* < 0.0001.

## 3. Results

### 3.1. Achromatic Color Schemes Preference in Different Crayfish Species

**Figure 1:**
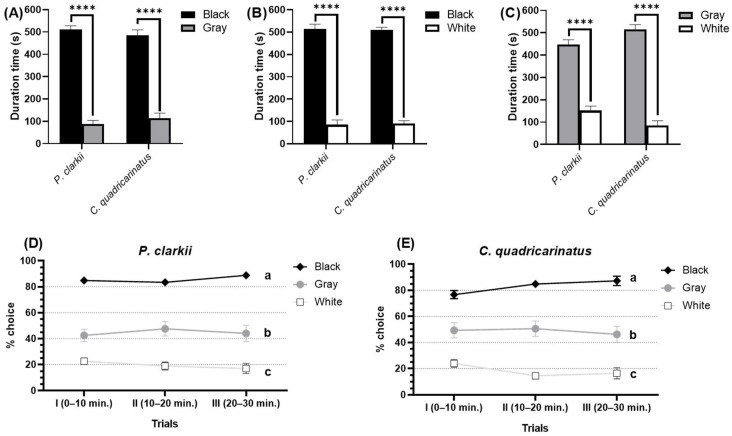
Achromatic color preference test in two different species of crayfish, *P. clarkii* and *C. quadricarinatus*. The effect of different achromatic colors—(**A**) black and white combination, (**B**) black and gray combination, and (**C**) gray and white combination—on crayfish color preference duration times. Percent of individuals, (**D**) *P. clarkii* and € *C. quadricarinatus*, making the choice of achromatic colors across trials. The data were displayed as means ± SEM, and statistical differences were analyzed using unpaired *t*-test (**A**–**C**) (**** *p* < 0.0001; *n* = 20) and two-way ANOVA with Tukey’s multiple comparison tests (**D**,**E**) (different letters a, b, c, and d indicate significant statistical differences with *p* < 0.05; *n* = 20).

Three achromatic colors, black, gray, and white, were used to observe the achromatic color preference in different crayfish species. Based on the results, both of the crayfish prefer to stay in black, followed by gray, then white (Figure 1). In comparison, a significant difference was shown between these three achromatic colors. When given a choice of black and white colors, both *P. clarkii* and *C. quadricarinatus* chose black over white and significantly spent a longer duration time in black (511.6 ± 2.666 s) than white area (88.22 ± 2.856 s) (*p* < 0.0001) (Figure 1A). Similarly, when offered black and gray, both crayfish still preferred the black color and spent a significantly longer duration time in black (499.0 ± 12.65 s) than the gray area (101.0 ± 12.66 s) (*p* < 0.0001) (Figure 1B). Between the gray and white colors, both crayfish significantly spent a longer duration in the gray area than white (*p* < 0.0001) (Figure 1C). Both species prefer black, the color with the lowest value tone (darkest). Value is the relative lightness or darkness of a color. In this result, crayfish preferred to stay in a dark and lower lux intensity area. Three successive outcomes have consistent choice percentages through three trials (0–10 min., 10–20 min., and 20–30 min.). For *P. clarkii*, the black has the highest choice percentage (85.75 ± 1.619%), then gray (44.73 ± 1.500%), and white has the least percentage of choices (19.51 ± 1.637%) (Figure 1D). Similar results from *C. quadricarinatus* were also displayed with black (82.94 ± 3.202%) as the most preferable color, followed by gray (48.75 ± 1.285%), then white (18.31 ± 2.857%) through all three consecutive trials (Figure 1E).

### 3.2. Standard Color Preference Test in Two Crayfish Species

**Figure 2:**
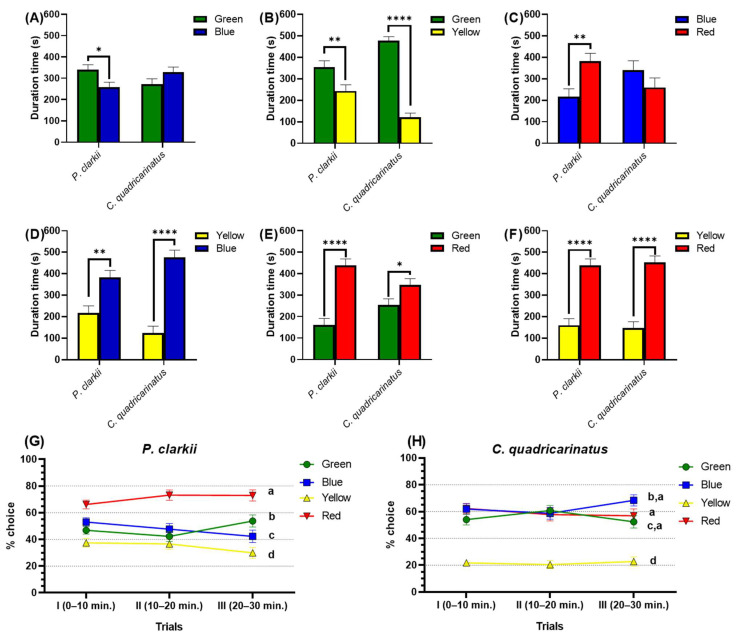
Four standard (red, green, blue, and yellow) color preference tests in two different species of crayfish, *P. clarkii* and *C. quadricarinatus*. The effect of different two-color combinations—(**A**) green and blue combination, (**B**) green and yellow combination, (**C**) blue and red combination, (**D**) blue and yellow combination, (**E**) green and red combination, and (**F**) red and yellow combination—on crayfish color preference duration times. Percent of individuals, (**G**) *P. clarkii* and (**H**) *C. quadricarinatus*, making the choice of different colors across trials. The data were displayed as means ± SEM, and statistical differences were analyzed using unpaired *t*-test (**A**–**F**) (* *p* < 0.05; ** *p* < 0.01; **** *p* < 0.0001; and *n* = 20) and two-way ANOVA with Tukey’s multiple comparison tests (**G**,**H**) (different letters a, b, c, and d indicate significant statistical differences with *p* < 0.05; *n* = 20).

Another test was conducted to further evaluate the chromatic color preference in crayfish. Four different colors (red, green, blue, and yellow) with two color combinations were applied as color substrates in the test tanks. Based on the results, *P. clarkii* manifests color preference with the ranking from most to least as red > green > blue > yellow. However, for *C. quadricarinatus*, a different color preference was displayed. There were no significant differences in color preference ranking between red, green, and blue. In addition, similar to *P. clarkia*, this species also avoided the color yellow (Figure 2). From the duration time of stay in two color combinations, *P. clarkii* spent a significantly longer time in green (341.4 ± 22.43 s) over blue (258.6 ± 22.42 s) (*p* = 0.0129); green (355.7 ± 28.50 s) over yellow (244.3 ± 28.50 s) (*p* =0.0088); red (382.6 ± 36.64 s) over blue (217.1 ± 36.67 s) (*p* = 0.0028); blue (382.5 ± 32.87 s) over yellow (217.5 ± 32.87 s) (*p* = 0.0011); red (439.0 ± 29.84 s) over green (161.0 ± 29.84 s) (*p* < 0.0001); and red (439.6 ± 29.89 s) over yellow (160.4 ± 29.89 s) (*p* < 0.0001). Through three trials (0–10 min.; 10–20 min.; and 20–30 min.), *P. clarkii* displayed consistent results, with red being the most preferable color and yellow being the least favorable color (Figure 2G). Meanwhile, in *C. quadricarinatus*, the choice percentage is mixed with green, blue, and red. However, similar to *P. clarkii*, yellow is also the lowest percentage choice of color for *C. quadricarinatus* (Figure 2H). Here, we notice that *P. clarkii* emphasizes its innate response in its interest in a color preference more than the *C. quadricarinatus*.

### 3.3. Color Preference in Juvenile American Crayfish

**Figure 3:**
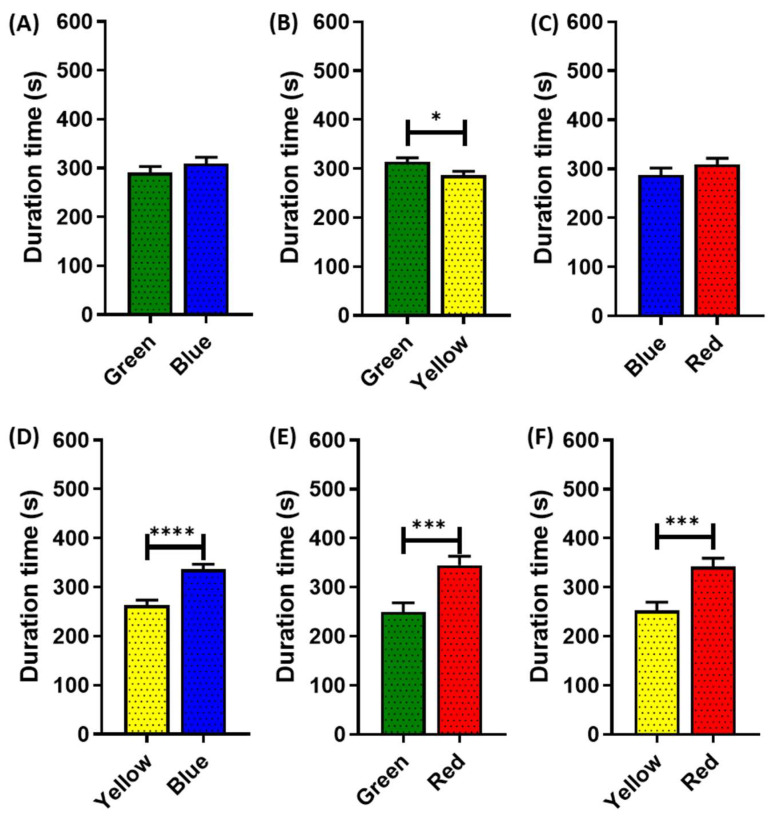
Four standard color preference tests in juvenile *P. clarkii* (~3 months old). The effect of different two-color combinations—(**A**) green and blue combination, (**B**) green and yellow combination, (**C**) blue and red combination, (**D**) blue and yellow combination, (**E**) green and red combination, and (**F**) red and yellow combination—on crayfish color preference duration times. The data were displayed as means ± SEM, and statistical differences were analyzed using unpaired *t*-test (* *p* < 0.05; *** *p* < 0.001; **** *p* < 0.0001; and *n* = 20).

Based on the promising results from the *P. clarkii* color preference, we were intrigued to conduct the same test in different life stages of this species. We applied for the color preference test in juvenile *P. clarkii*. Similar to the adult of *P. clarkii*, the juvenile ones mostly favored red and avoided yellow (Figure 3). When introduced to the green and red color combinations, they preferred staying in the red area for a significantly longer duration (*p* = 0.0006) (Figure 3E). Meanwhile, using yellow color combinations, yellow always has a significantly shorter duration time, either paired with the green, blue, or red colors (Figure 3B,D,F). However, there is no significant difference in the duration of juvenile crayfish spent on green–blue (*p* = 0.2968) and blue–red (*p* = 0.2848) partitions. These results might happen because at the juvenile level, *P. clarkii* are still in development; they are not yet mature and tend to explore more areas.

**Figure 4:**
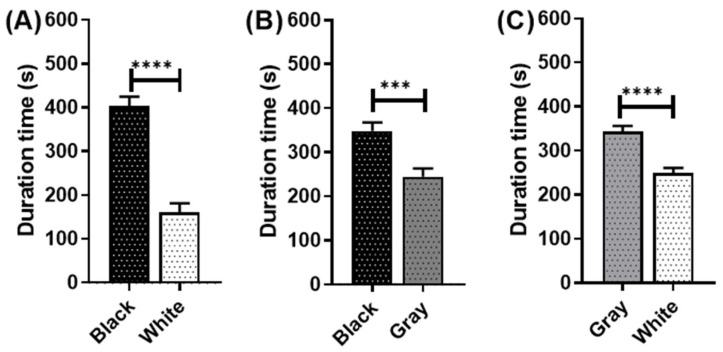
Achromatic color preference test in juvenile *P. clarkii*. The effect of different achromatic colors—(**A**) black and white combination, (**B**) black and gray combination, and (**C**) gray and white combination—on crayfish color preference duration times. The data were displayed as means ± SEM, and statistical differences were analyzed using unpaired *t*-test (*** *p* < 0.001; **** *p* < 0.0001; and *n* = 20).

Meanwhile, for the achromatic color preference, the juvenile *P. clarkii* displayed consistent results with the adult one. The color ranking is black > gray > white. The black is the most preferable color. When introduced to the black and white combination (Figure 4A), the juvenile significantly stayed longer in black (403.0 ± 21.57 s) than in the white color (160.9 ± 20.38 s) (*p* < 0.0001). The juvenile also has a significantly longer duration time (*p* = 0.0003) in black (348.5 ± 19.11 s) over the gray (244.7 ± 18.54 s) color (Figure 4B). They also chose gray over white, as observed by a significantly higher duration time (*p* < 0.0001) in the gray (343.2 ± 13.05 s) than white area (248.6 ± 12.23 s) (Figure 4C).

### 3.4. Color Preference in American Crayfish of Different Sexes

**Figure A2:**
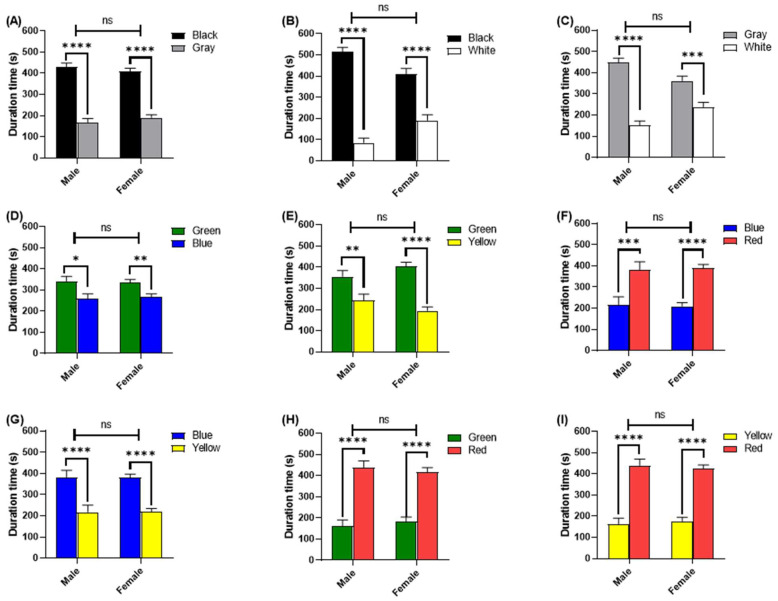
Color preference tests between males and females of *P. clarkii*. The color partition of two-color combinations—(**A**) black and gray, (**B**) black and white, (**C**) gray and white, (**D**) green and blue, (**E**) green and yellow, (**F**) blue and red, (**G**) blue and yellow, (**H**) green and red, and (**I**) red and yellow—was applied on crayfish color preference duration time test. The data were displayed as means ± SEM, and statistical differences were analyzed using unpaired *t*-test for color combination and nested *t*-test for male–female comparison (* *p* < 0.05; ** *p* < 0.01; *** *p* < 0.001; **** *p* < 0.0001; ns = not significant; and *n* = 20).

We also compare the different gender of *P. clarkii* to observe whether there are color preference differences in male or female crayfish. Our hypothesis is male and female crayfish may exhibit differential preferences for certain colors, potentially driven by behavioral or ecological reasons. However, based on the result, there is no significant difference between male and female crayfish in color preference (Figure A2). Similar color preference ranking was displayed from both male and female crayfish. In achromatic colors, black is the most preferred color and white is the least preferred color (Figure A2A–C). For the standard color ranking as follows: red > green > blue > yellow. Due to no significant difference of duration time from male and female in a color preference test, we decided to use a mix of male and female crayfish for further experiment.

### 3.5. Color Preference Alterations after Antidepressant Exposure

**Figure 5:**
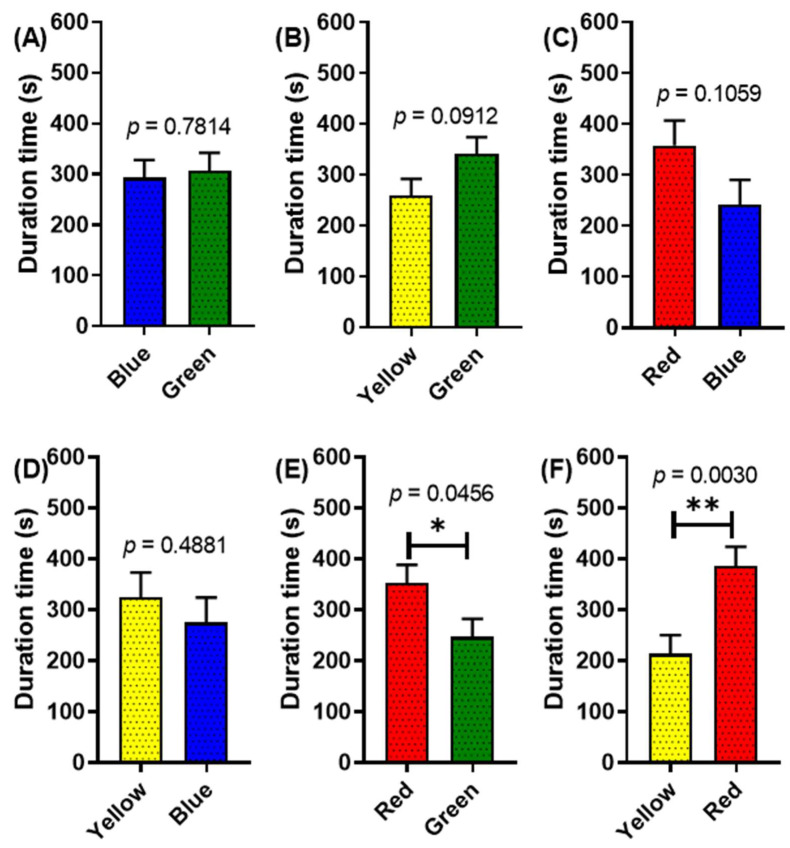
Four standard color preference tests in *P. clarkii* after 14 days of exposure with sertraline 1 µg/L. The effect of different two-color combinations—(**A**) green and blue combination, (**B**) green and yellow combination, (**C**) blue and red combination, (**D**) blue and yellow combination, (**E**) green and red combination, and (**F**) red and yellow combination—on crayfish color preference duration times. The data were displayed as means ± SEM, and statistical differences were analyzed using unpaired *t*-test (* *p* < 0.05; ** *p* < 0.01; and *n* = 12).

In addition, we evaluated the crayfish color preference toward the potential adverse effect of the antidepressant sertraline. After exposure to sertraline 1 µg/L for 14 days, the innate color preference in *P. clarkii* was disrupted. In normal conditions, yellow is the least preferable color to the other three colors (red, green, and blue). However, in these results, the crayfish spent more time in the yellow partition than the blue (although there is no statistical difference) (Figure 5D). There is also no strong color preference (no statistical differences) displayed in the other color partitions, such as blue–green; yellow–green; and red–blue (Figure 5A–C). However, when introduced to red–green and red–yellow, significant differences in duration time were displayed. *P. clarkii* significantly spent more time in the red partition than the green partition (*p* = 0.0456) (Figure 5E). Furthermore, when put in a red–yellow partition, they also spent a significantly longer duration time in the red than in the yellow color (*p* = 0.0030) (Figure 5F). Based on this result, it seems antidepressants have adverse effects on this animal model that might disrupt their ability to differentiate colors. Despite that, these crayfish still have a strong preference toward the red color.

**Figure 6:**
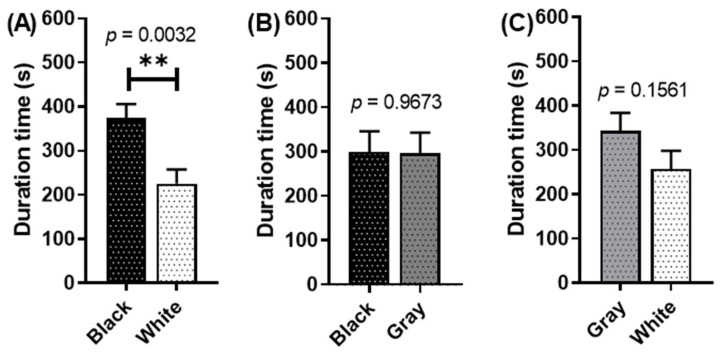
Achromatic color preference tests in *P. clarkii* after 14 days of exposure with sertraline of 1 µg/L. The effect of different two-color combinations—(**A**) black and white combination, (**B**) black and gray combination, and (**C**) gray and white combination—on crayfish color preference duration times. The data were displayed as means ± SEM, and statistical differences were analyzed using unpaired *t*-test (** *p* < 0.01; and *n* = 12).

The antidepressant exposure also disrupts the crayfish color preference in achromatic colors. Based on the black and white color partition, the crayfish still prefer black over white (Figure 6A). It seems the crayfish are still able to distinguish these contrasting colors that differ from one another. Meanwhile, for other colors with low, different amounts of tint and shade (black–gray or white–gray), the sertraline-exposed crayfish did not show any significant difference in duration time. In the black–gray color partition, there was no significant difference in the duration time (Figure 6B). The same was applied when the sertraline-exposed crayfish were introduced in a gray and white color partition, and no significant difference in duration time was displayed (Figure 6C). In addition, sertraline was also able to alter the crayfish’s behavior. In several color partitions, the sertraline-treated crayfish have a tendency to explore more of the area, as displayed by the significantly higher number of switching between different colors than the control (Table A3).

## 4. Discussion

It was reported that crustaceans have a large variety of eye types. Some have simple eyes like humans; compound eyes similar to insects; eyes with mirrored optics; and eyes without optics [30]. Mantis shrimps (*Stomatopods*) are known as crustaceans with polychromatic vision with 12 cone types of color receptors in their compound eye, supporting them to decode the color information [31]. Even so, we still have no idea how they perceive colors or the mechanism behind them. Others suggested that despite the mantis shrimp having plenty of photoreceptors, it surprisingly displayed poor performance in color discrimination [32]. Another crustacean, such as the blue crab (*Callinectes sapidus*), displayed their courtship and mate choice based on the color cues. The male blue crab has a preference to choose females with red claw dactyls [33]. The larva of freshwater prawns (*Macrobrachium rosenbergii*) has a color preference for blue [34]. Meanwhile, a crayfish consists of two compound eyes with photoreceptor neurons sensitive to light [18]. The functional role of those photoreceptors in the color vision of crayfish has not been adequately examined. It was reported that crayfish can only see at least two colors, blue and red [35]. Nosaki and colleagues confirmed the crayfish’s eye has at least two photoreceptors that are selectively sensitive to two different spectral regions (460 and >600 nm) [36]. No recent discussion can be found to further evaluate this finding. Most of the studies about crayfish’s innate color preference referred to dark (black) or light (white) preference [37,38,39,40]. Crayfish, also called crawfish, are crustaceans; most live in freshwater, and few live in brackish or salt water. Most of the freshwater crayfish live in burrows, lakes, and rivers. They tend to hide in the darkness under rocks or logs during the daytime and become more active at night. The crayfish’s dark–light preference tests further evaluated this habit in a plus maze. It revealed that noble crayfish (*Astacus astacus*) avoid the light arm of the maze [41]. Other research also showed that crayfish naturally explore new environments but generally prefer dark places [42]. A black and white color preference test for crayfish has also been tested, which revealed that crayfish prefer to stay on the black substrate [43].

Based on the results, *P. clarkii* and *C. quadricarinatus* have slightly different innate color preferences. *P. clarkii* strongly prefers red, followed by green, blue, then yellow. Meanwhile, *C. quadricarinatus* did not strongly prefer specific colors, but the least preferable color is yellow. So far, no studies specifically evaluate the innate color preferences of these two species. However, crayfish tend to stay in an environment with similar vibes to their exoskeleton color [44]. Specific color patterns help crayfish conceal themselves from predators or potential prey they seek. A crayfish’s exoskeleton is called a carapace. Crayfish bodies are covered in this hard protective layer, which protects them from predators and other environmental threats. A carapace is made of several layers of chitin, a tough fibrous material [45]. The color of a crayfish’s carapace was determined in response to the interaction between genetics and their environment [46]. For example, when crayfish are exposed to cold water, they often turn a shade of gray or brown. This phenomenon is known as “thermal melanism” and is thought to be the crayfish’s adaptation to help them better blend in with their surroundings [47,48]. In terms of achromatic color preference, both *P. clarkii* and *C. quadricarinatus* strongly prefer the color black. According to previous studies, crayfish tend to stay in dark environments and avoid direct light [49,50]. In different life stages, juvenile crayfish tend to have less color preferences than the adults as displayed in our result. A similar result was displayed by Beingesser and Copp, 1985, that showed juvenile crayfish appear cryptic against the background color more than the adults [51]. This happened because juvenile crayfish remain more active than adults [52]. Juvenile crayfish are also more likely to react to a stimulus than adults. They prefer to avoid potential predators than to maintain their ground, which represents their adaptive response toward predation risk [53]. In sexual difference, there is no difference in color preference in males and females. In our knowledge, there is no study that specifically evaluates the color preference in different sex of crayfish. A study shows another crustacean: the male blue crab prefers females with red claws than yellow or orange claws [54]. It appears that the color preference in either males or females is affected based on their maternal and offspring behaviors [37]. Similarly, crayfish are also under intense sexual selection pressure in their mating systems. Their mating selection is also affected by the color of exoskeleton and claws [55]. Despite their picky selection, which involves coloration, individual male or female crayfish showed no different color preference when introduced to the novel environment. Up to now, this is the first study to report similarities in color perception between male and female crayfish.

Color preferences have been linked with depression and anxiety in humans, and these changes have already been used to determine mental states [56]. Another aquatic animal, the zebrafish, has a color preference ranking pattern as blue > red > green > yellow. In a previous study, it was shown that zebrafish treated with sertraline showed a reduced choice index in the red color, which switches the color preference ranking into blue > green > red > yellow [57]. In a similar case, in this study, the sertraline-treated crayfish also displayed a lower choice index when the green or blue colors were introduced. It seems the antidepressant is unable to suppress the strong color preference of crayfish toward the color red (the first color ranking) but was still able to disrupt the second-in-line for color ranking, like blue or green (second or third ranking). The disruption in this color preference happens because of the neurobehavioral side effects caused by antidepressants [58]. It affects the vision and color cues essential in guiding the animal’s behavior [59,60]. The use of sertraline for an extended period can affect the levels of the neurotransmitter serotonin in the brain, which also results in anxiety and depression [42]. Evidence shows that changes in emotional states can affect how people perceive color [61]. Subjects with depression exhibit a reduced ability to distinguish colors, resulting in a fuzzy and less vivid view [62]. Another explanation is that the antidepressant also alters the crayfish behavior, which further affects various response metrics. In a previous study, it was reported that crayfish exposed to sertraline were more aggressive and displayed bold behavior with higher locomotion activity [63,64]. Crayfish exposed to sertraline also spent more time outside the shelter, which indicates less stress and less preference for a dark color area [65]. This combination of antidepressant effects that affect vision and behavior is what ultimately causes the crayfish color preferences to be disrupted. The color preference assay was demonstrated in this study as a potential tool for assessing chemical toxicology in a behavioral context. Nevertheless, further studies need to be conducted to understand the mechanism of antidepressants that affect color preference in this aquatic animal model. Considering that this antidepressant is one of the highest water pollutant contributors, it is important to evaluate the hazard of this compound to the survival of aquatic animals.

## 5. Conclusions

Crustaceans, like crayfish, have evolved various types of eyes to perceive colors in their environment. Yet, it is unclear how they perceive or decode color information. Even though crustaceans have a variety of photoreceptors, not all are effective at discriminating colors. In this study, both *P. clarkii* and *C. quadricarinatus* show a strong preference for the color black. Their preference hierarchy is consistent as black > gray > white. Consequently, crayfish prefer dark and low-lux areas, possibly reflecting their natural habitats or instinct to avoid predators. Color preference in *P. clarkii* appears to be more defined than in *C. quadricarinatus*. *P. clarkii* prefers red and avoids yellow in both the juvenile and adult stages. However, juveniles showed no significant preference for some color combinations, suggesting they might be more exploratory due to their developmental stage. Both sexes have similar rankings in achromatic and standard colors. Further, post-antidepressant exposure altered crayfish preferences, especially for intermediate colors. Red remained their most preferred color, but crayfish’s ability to differentiate colors with similar tints and shades was significantly affected. It indicates a potential impairment in color vision due to the antidepressant drug side effects. The present study provides valuable insights into crayfish color preferences and emphasizes the potential behavioral effects of pollutants on this animal model.

## Data Availability

Data will be made available on request.

## Appendix A

**Table A1:** Summary of previous knowledge regarding innate color preference in crustaceans.

Species	Color Preference	Instrumental Setting	Reference
*Litopenaeus vannamei*	*L. vannamei* prefer blue color pellets	Determine the involvement of color vision by food color preference	Kawamura et al., 2017 [66]
*Callinectes sapidus*	Males significantly preferred red-clawed females to orange-clawed females	Behavioral experiments evaluating male color preference toward photographs of female crabs with sexually receptive posture	Baldwin, J and Johnsen, S., 2012 [54]
*Eriocheir sinensis*	The crabs showed no food color preference	The food color preference of *E. sinensis* was observed using several foods dyed in different colors	Chen et al., 2023 [67]
*Macrobrachium rosenbergii*	The number of larvae was highest around the dark blue, light blue, and white beads; lowest around the black, red, and light green beads.	Plastic beads (4.1 mm in diameter) of different colors (dark blue, light blue, light green, yellow, red, white, black, and gray) in various combinations	Kawamura et al., 2016 [34]
*Pacifastacus leniusculus*	Crayfish were not attracted by the light, which was indicated by the lower catches in traps	Catch per unit effort (CPUE) comparison with white and green LED lights attached inside the baited traps or baited traps without light	Roukonen et al., 2021 [68]
*Cambarus chasmodactylus* and *Orconectes cristavarius*	*O. cristavarius* sheltering behavior significantly increased when exposed to both cool and warm spectrum LEDs at 2000 h.	Exposed those crayfish to cool (5000 K)- and warm (3000 K)-spectrum LED lights at ecologically relevant intensities of 15 lx in a laboratory experiment	Fischer, J. R., 2016 [69]
*Procambarus clarkii* and *Orconectes australis*	Crayfish have a statistically significant preference for a black- colored bottom over white	The black and white substrates preference experiment	Bierbower, S. M., 2010 [70]

**Table A2:** Wavelength (spectrum), irradiance (emission intensity), and reflectance of all color plates.

Color	Wavelength Spectrum (nm) and Maximum Wavelength	Irradiance (uWatt/nm)	Reflectance (%)
White	380–700 (561)	0.000691	100
Gray	400–700 (564)	0.000207	96
Black	500–700 (588)	0.0000342	80
Blue	450–485 (456)	0.000358	98
Green	500–565 (541)	0.000376	100
Yellow	565–590 (572)	0.000587	99
Red	625–740 (625)	0.000489	97

**Table A3:** The comparison number of switching between different colors in control and sertraline-treated groups (* *p* < 0.05, ** *p* < 0.01, *** *p* < 0.001, and n.s. = not significant).

Colors	Number of Switching between Different Colors (Means ± SEM)		t Value	*p* Value	Significance
	Control	Sertraline			
Green–Blue	16.92 ± 2.317	23.75 ± 4.331	1.391	0.1781	n.s.
Green–Yellow	14.50 ± 1.960	21.00 ± 3.174	1.742	0.0954	n.s.
Blue–Red	15.25 ± 2.980	19.33 ± 4.809	0.7218	0.4780	n.s.
Yellow–Blue	11.50 ± 2.337	28.17 ± 7.486	2.125	0.0450	✱
Green–Red	13.00 ± 2.146	23.58 ± 3.201	2.746	0.0118	✱
Yellow–Red	12.00 ± 2.629	30.00 ± 3.155	4.383	0.0002	✱✱✱
Black–White	17.33 ± 6.844	58.67 ± 10.00	3.410,	0.0025	✱✱
Gray–White	13.50 ± 1.952	38.83 ± 12.46	2.009	0.0570	n.s.
Black–Gray	8.583 ± 2.017	46.58 ± 10.05	3.707	0.0012	✱✱

**Table A4:** The *t* and *p* values from unpaired *t* test of color preferences from different groups (* *p* < 0.05, ** *p* < 0.01, *** *p* < 0.001, **** *p* < 0.0001, and n.s. = not significant).

Color Preference (Color 1 vs. Color 2)	Group	Difference Between Means ± Standard Error (Color 2—Color 1)	t Value	*p* Value	Significance
**Green vs. Blue**	*P. clarkii*	−82.73 ± 31.71	2.609	0.0129	✱
	*C. quadricarinatus*	55.37 ± 35.25	1.571	0.1245	n.s.
	Females	67.31 ± 21.62	3.113	0.0035	✱✱
	Males	75.55 ± 32.18	2.348	0.0242	✱
	Juvenile	18.91 ± 17.92	1.055	0.2968	n.s.
	Sertraline	−14.01 ± 49.86	0.2809	0.7814	n.s.
**Green vs. Yellow**	*P. clarkii*	−111.4 ± 40.31	2.764	0.0088	✱✱
	*C. quadricarinatus*	355.8 ± 26.59	13.38	<0.0001	✱✱✱✱
	Females	−210.4 ± 25.53	8.243	<0.0001	✱✱✱✱
	Males	−121.0 ± 39.57	3.058	0.0041	✱✱
	Juvenile	−27.58 ± 12.06	2.287	0.0269	✱
	Sertraline	−82.47 ± 46.69	1.766	0.0912	n.s.
**Blue vs. Red**	*P. clarkii*	165.5 ± 51.84	3.193	0.0028	✱✱
	*C. quadricarinatus*	−79.76 ± 62.72	1.272	0.2112	n.s.
	Females	180.6 ± 24.25	7.446	<0.0001	✱✱✱✱
	Males	188.0 ± 43.37	4.333	0.0001	✱✱✱
	Juvenile	20.80 ± 19.22	1.082	0.2848	n.s.
	Sertraline	116.5 ± 69.10	1.686	0.1059	n.s.
**Yellow vs. Blue**	*P. clarkii*	164.9 ± 46.49	3.548	0.0011	✱✱
	*C. quadricarinatus*	353.5 ± 46.91	7.536	<0.0001	✱✱✱✱
	Females	−163.0 ± 22.08	7.382	<0.0001	✱✱✱✱
	Males	−214.3 ± 40.85	5.246	<0.0001	✱✱✱✱
	Juvenile	72.92 ± 15.26	4.777	<0.0001	✱✱✱✱
	Sertraline	−49.10 ± 69.62	0.7052	0.4881	n.s.
**Green vs. Red**	*P. clarkii*	277.9 ± 42.20	6.587	<0.0001	✱✱✱✱
	*C. quadricarinatus*	93.41 ± 41.77	2.236	0.0313	✱
	Females	233.4 ± 30.00	7.779	<0.0001	✱✱✱✱
	Males	280.5 ± 41.76	6.716	<0.0001	✱✱✱✱
	Juvenile	94.93 ± 25.75	3.686	0.0006	✱✱✱
	Sertraline	105.8 ± 49.94	2.120	0.0456	✱
**Yellow vs. Red**	*P. clarkii*	279.2 ± 42.28	6.604	<0.0001	✱✱✱✱
	*C. quadricarinatus*	305.9 ± 42.01	7.281	<0.0001	✱✱✱✱
	Females	246.8 ± 26.16	9.437	<0.0001	✱✱✱✱
	Males	325.7 ± 34.12	9.544	<0.0001	✱✱✱✱
	Juvenile	89.31 ± 24.89	3.589	0.0008	✱✱✱
	Sertraline	173.7 ± 52.19	3.329	0.0030	✱✱
**Black vs. White**	*P. clarkii*	−428.9 ± 30.41	14.11	<0.0001	✱✱✱✱
	*C. quadricarinatus*	−417.8 ± 18.11	23.08	<0.0001	✱✱✱✱
	Females	−218.7 ± 37.72	5.796	<0.0001	✱✱✱✱
	Males	−459.9 ± 18.57	24.77	<0.0001	✱✱✱✱
	Juvenile	−242.1 ± 29.68	8.159	<0.0001	✱✱✱✱
	Sertraline	−148.0 ± 44.82	3.303	0.0032	✱✱
**Gray vs. White**	*P. clarkii*	−296.9 ± 28.38	10.46	<0.0001	✱✱✱✱
	*C. quadricarinatus*	−430.3 ± 29.82	14.43	<0.0001	✱✱✱✱
	Females	−124.0 ± 31.74	3.907	0.0004	✱✱✱
	Males	−340.0 ± 16.90	20.12	<0.0001	✱✱✱✱
	Juvenile	−94.56 ± 17.89	5.286	<0.0001	✱✱✱✱
	Sertraline	−85.64 ± 58.31	1.469	0.1561	n.s.
**Black vs. Gray**	*P. clarkii*	−423.4 ± 22.67	18.68	<0.0001	✱✱✱✱
	*C. quadricarinatus*	−372.7 ± 32.35	11.52	<0.0001	✱✱✱✱
	Females	−219.5 ± 19.36	11.34	<0.0001	✱✱✱✱
	Males	−262.4 ± 23.94	10.96	<0.0001	✱✱✱✱
	Juvenile	−103.8 ± 26.63	3.900	0.0003	✱✱✱
	Sertraline	−2.707 ± 65.22	0.04151	0.9673	n.s.
